# Study on Extraction and Purification of *Acanthopanax senticosus* Polyphenols by an Ionic Liquid-Assisted Aqueous Two-Phase System

**DOI:** 10.3390/molecules28176383

**Published:** 2023-08-31

**Authors:** Ying Li, Xiaoli Li, Xueyan Wang, Jiaojiao Xue, Rui Zhang, Yi Ding, Xiuling Chu, Jianqing Su

**Affiliations:** Shandong Provincial Institute of Chinese Veterinary Medicine, Liaocheng University, Liaocheng 252000, China; ly15963373832@163.com (Y.L.); lxl15006995983@163.com (X.L.); wangxueyan202203@163.com (X.W.); x15065441211@163.com (J.X.); m17863709708@163.com (R.Z.); djy15373023047@163.com (Y.D.)

**Keywords:** ionic liquids, aqueous two-phase systems, *Acanthopanax senticosus*, polyphenols

## Abstract

This study aimed to extract and purify polyphenols from *Acanthopanax senticosus*. A new green method was developed, in which ionic liquids (ILs) were used as aqueous two-phase (ATP) adjuvants to extract the polyphenols from *A. senticosus*. An ionic liquid-assisted aqueous two-phase system (IL-ATPS) was established. The purification of the polyphenols from the extraction fluid by AB-8 macroporous resin was conducted, and the kinetic mechanisms were studied. The reuse of ionic liquids was executed. The results showed that an [OMIM]Br-assisted ethanol/NaH_2_PO_4_ system (IL-ATPS) was the best extraction solvent. In this study, the following optimal extraction conditions were determined: 32 wt.% ethanol, 25 wt.% NaH_2_PO_4_, 9 wt.% additional ionic liquid, a solid–liquid ratio of 1:40 g/mL, an extraction temperature of 50 °C, a pH of 4.0, an extraction time of 50 min, and an extraction rate of the polyphenols at 15.90 mg/g. The optimum adsorption parameters of the macroporous resin AB-8 were as follows: a flow rate of 3.5 BV·h^−1^, a sample volume of 40 mL, an elution flow rate of 3.5 BV·h^−1^, an eluent volume of 80 mL, and an eluant that was constituted by an 85% volume fraction of ethanol. The decolorization effect of 4% activated carbon was better than the other amounts; in addition, a decolorization rate of 76.81% and an ionic liquid recovery rate of 81.12% were found to be the most optimal. Compared with the traditional extraction methods, IL-ATPS has the advantages of requiring simple operation, saving time, and high efficiency. In addition, it can be used for the extraction of the polyphenolic compounds.

## 1. Introduction

*Acanthopanax senticosus* (AS) is a homologous food and medicine plant that is used in clinical settings, healthcare, and has an extensive history in China [1]. The polyphenols from AS are one of the noted pharmacologically active substances [2]. They have antioxidant [3], anti-inflammatory [4], antibacterial [5], and anti-enzymatic [6] properties. Their clinical applications are based on the extraction and purification of polyphenols. The selection of the extraction method and solvent used is related to the extraction efficiency and biological activity of the polyphenols. There are various extraction methods for the polyphenols, including solvent extraction, ultrasonic-assisted extraction, microwave-assisted extraction, and enzyme extraction, among which ultrasonic extraction has the advantages of a high extraction efficiency, simple operation, safety, a significantly shortened extraction time, and not destroying the active components.

The traditional polyphenol extraction solvents that are mostly used are ethanol, acetone, and other organic solvents. These organic solvents present flammability, volatility, toxicity, and other such shortcomings, and their use can easily cause personnel and environmental harm. Ionic liquids (ILs) have good stability, are not volatile, and possess an ecological friendliness when compared with conventional organic solvents [7]. ILs can be regarded as a substitute for traditional volatile solvents in the processes of organic synthesis, chemical separation, and the extraction of target components. Ionic liquids have been successfully applied to extract various active substances from plants, such as coumarins, terpenoids, phenylpropanoids, and flavonoids [8].

The aqueous two-phase (ATP) method is a new green extraction method that is widely used to extract active plant ingredients. It has an excellent performance in terms of being low cost and having scalability, a capacity for continuous operation, and environmental friendliness [8]. This study showed that an IL added to an aqueous two-phase system (ATPS) can adjust the physical and chemical properties of upper and lower phases, and it can improve the extraction and application performance [6,9]. In the study by Joao et al. [10], IL-based aqueous biphasic systems (IL-ABS) and more traditional salt/polymer-based ABS were tested for their ability to extract eugenol and propyl gallate. It was found that adding ionic liquids can improve the extraction efficiency of an aqueous two-phase system. Ran et al. [11] added 5 wt.% [C6mim]BF4 into an ATPS, and the extraction efficiencies (EEs) of pro-anthocyanidins were dramatically improved from 47.89% to 52.42% and 97.79%. In addition, the amount of organic solvent needed was also reduced, which thus proved the excellent adjuvant characteristics of IL when combined with an aqueous two-phase system.

In this paper, we established a new method for extracting polyphenols by IL-ATPS, as well as optimized the extraction conditions via PB experiments and the response surface methodology. A macroporous resin was used to recover the polyphenols from the extraction, and the kinetics and thermodynamics of the recovery were studied. Finally, the reuse of ionic liquid was studied. Compared with ultrasonic-assisted ethanol extraction, this method has obvious advantages, such as a shorter extraction time: IL-ATPS was 40 min, ultrasonic-assisted was 2 h, less solvent usage: IL-ATPS was 32 wt.%, ultrasonic-assisted was 60 wt.%, less energy consumption: IL-ATPS was 395 W, ultrasonic-assisted was 420 W, and a higher extraction rate: IL-ATPS was 7.28%, ultrasonic-assisted was 2.08%. This research lays a foundation for the green extraction of plant polyphenols, as well as for the clinical application of *A. senticosus* polyphenol extraction.

## 2. Results and Discussion

### 2.1. Screening of the Aqueous Two-Phase System

**Figure 1:**
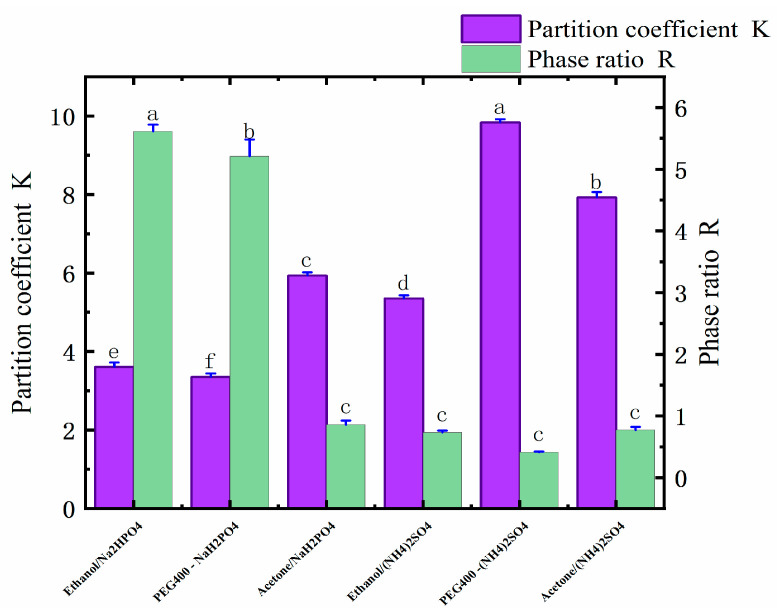
Distribution coefficients (K) and phase ratios (R) of six different ATPSs. Different letters represent significant differences (*p* < 0.05) between different vertical bars.

Six ATPSs were constructed that were composed of ethanol, water, (NH_4_)_2_SO_4_, and NaH_2_PO_4_; PEG400, water, (NH_4_)_2_SO_4_, and NaH_2_PO_4_; and acetone, water, (NH_4_)_2_SO_4_, and NaH_2_PO_4_. The ingredients are shown in Table 1. In the ATPSs, the upper and lower phases were formed by the substances in the upper phase and by salt competing for water molecules. As shown in Table 1, ethanol, PEG400, acetone, (NH_4_)_2_SO_4_, and NaH_2_PO_4_ all have good phase-forming abilities. The aqueous two-phase system formed by different types of salts has distinct physicochemical properties. Through the interaction of hydrogenic, ionic, and hydrophobic bonding, other aqueous two-phase systems have different affinities for different substances; thus, different active substances can be selectively extracted. As shown in Table 2 and Figure 1, when the type of salt was (NH_4_)_2_SO_4_, the yield of the polyphenols was PEG > ethanol > acetone, and the highest yield was 4.84 mg/g. When the salt was NaH_2_PO_4_, then the best yield of polyphenols followed the order of PEG400 > ethanol > acetone, and the highest yield was 5.66 mg/g. In an ATPS of acetone/salt, the low yield of the polyphenols may be due to the weaker affinity between acetone and polyphenols, as well as due to the low polarity of the salt in the lower phase, i.e., after the acetone and salt form the ATP, more suitable conditions for the lower-phase polyphenols’ molecules arise. From the partition coefficient, it can be seen that the partition coefficient of an acetone-formed aqueous two-phase system was 10.23 for acetone/NaH_2_PO_4_, and 20.66 for acetone/(NH_4_)_2_SO_4_. These coefficients are much higher than those found in the other systems, which may be due to the more active chemical properties of acetone (which also cannot stably bind polyphenol molecules, thus resulting in a low yield of polyphenols). The ethanol/NaH_2_PO_4_ aqueous two-phase system was finally selected for the follow-up test by comparing the yield of polyphenols and due to the adverse effect of the ammonium sulfate applied in the industry on the environment.

### 2.2. Selection of Ionic Liquids

The structures and attributes of the ILs significantly influenced the extraction efficiency of the point compounds. The hydrophilic and hydrophobic properties, hydrogen-bonding acidity/basicity, and chain length can be used as essential properties in the preliminary selection of IL species. The anions and cations of IL determined these properties. Nine imidazolium-based ILs ([C_4_mim]Cl, [C_4_mim]Br, [C_4_mim]BF_4_, [BMIM]OTF, [BMIM]SO_4_, [HMIM]Cl, [HMIM]BF_4_, [OMIM]BF_4_, and [OMIM]Br) were investigated in this study as adjuvant-assisted ethanol/NaH_2_PO_4_ systems, which were then used to extract polyphenols. The optimal ionic liquid was screened with the yield of the polyphenols. The results are provided in Figure S1 and Table S1.

Imidazolium-based ILs can interact with ethanol, mainly via H-bonding (between the hydrogen atoms of the imidazolium ring (specifically C-H)), as well as via the length of the alkyl chain of imidazole-based ILs, which can change the polarity of ethanol [12]. These inter-reactions may shield ethanol from the circumferent water molecules, thus inducing the exclusion of ethanol by the kosmotropic salt. This trend is an outcome of enhancing the fluid’s overall hydrophobicity of the IL, which is due to the increase in the alkyl chain length of the IL (alkyl chain C < 6). These properties also support the phase separation. Hydrochloric acid noticeably enhanced the water uptake and aqueous IL solubility, and the amount of IL cations dissolved in the acidic aqueous phase was higher than that of the anions [13]. The salt solution of the lower phase was NaH_2_PO_4_, which made the whole system acidic and promoted the dissolution of ionic liquids, especially for ionic liquids with long alkyl chains. There was a similar hydrophobicity in the parameters of the cations, but the water solubility data of their anions still diverged [14]. As a result, the ATPS formation was made easier. When the alkyl chain of IL is better than the six carbons, or when it is more significant than its corresponding critical micelle concentration (CMC) [15], then the ionic liquid will self-aggregate. However, imidazole-based ILs can increase their solubility in water after self-aggregation, thus weakening the hydrophobic effect on ATPS. Due to the H-bonding and hydrophobic interplay between imidazolium-based ILs and ethanol, ethanol will react with the ionic liquid to form a complex. Thus, almost all ILs exist in the ethanol phase. Therefore, the polarity of the upper phase can be enhanced by increasing the length of the IL alkyl chain, and the two phases’ polarity difference can be enhanced such that the phase range of ATPS can be broadened.

**Figure 2:**
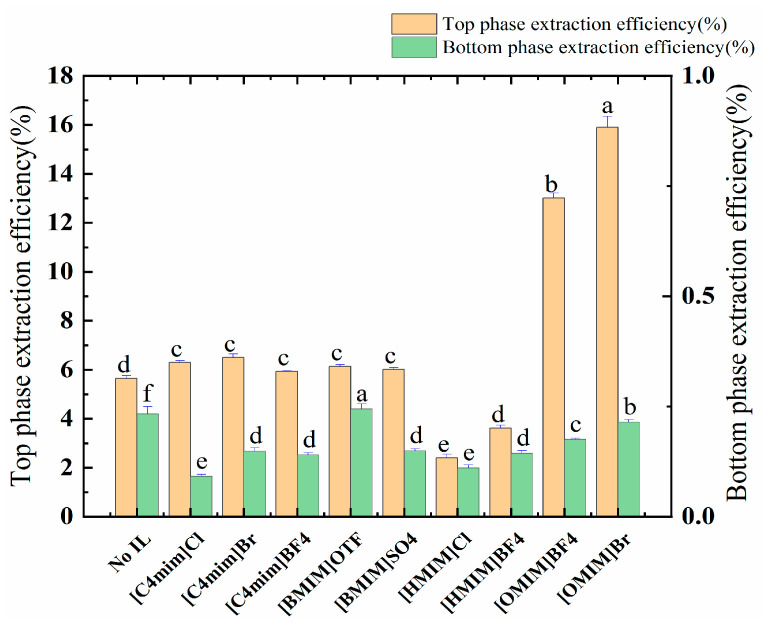
The effect of ILs on the extraction rate of the polyphenols of the top and bottom phases. Different letters represent significant differences (*p* < 0.05) between different vertical bars.

The allocation of polyphenols between an ATPS is an elaborate phenomenon, and it is guided mainly by the interplay of the allocated solute and the phase ingredients, e.g., through hydrogen bonding, van der Waals forces, hydrophobic and electrostatic interactions, as well as steric and conformational effects. Due to the presence of the ionic liquid, the electrostatic interaction, van der Waals force, and hydrogen bonding force can enhance the extraction rate of the polyphenols. The data provided in Figure 2 and Table 3 show that [OMIM]BF_4_ and [OMIM]Br (alkyl chain carbon atom > 6) have significant effects on the distribution of the target molecules. This process also increased the partition coefficient of the ATPSs from 7.04 to 26.719 and 25.337; in addition, the polyphenol yield increased from 5.66 mg/g to 13.01 mg/g and 15.90 mg/g. Finally, [OMIM]Br was selected as the adjuvant by comparing the yields of polyphenols.

### 2.3. Single-Factor Effects on IL-ATPS

**Figure 3:**
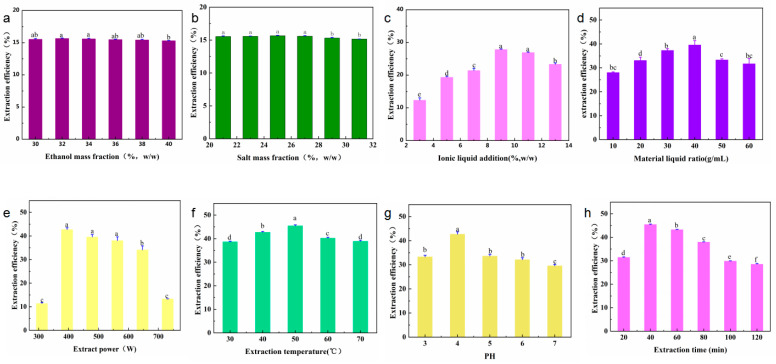
Single factors affecting the yield of *A. senticosus* polyphenols: (**a**) ethanol mass fraction (21~31 wt.%), (**b**) salt mass fraction (30~40 wt.%), (**c**) ionic liquid addition (3~13 wt.%), (**d**) material–liquid ratio (1:10~1:60 g/mL), (**e**) extraction power (311~731 W), (**f**) extraction temperature (30~70 °C), (**g**) pH (3~7), and (**h**) extraction time (20~120 min). Different letters represent significant differences (*p* < 0.05) between different vertical bars.

This study analyzed the mass fraction of salt (21, 23, 25, 27, 29, and 31 wt.%), mass fraction of ethanol (30, 32, 34, 36, 38, and 40 wt.%), mass fraction of ionic liquids (3, 5, 7, 9, 11, and 13 wt.%), the solid–liquid ratio of the IL-ASTPs (1:10, 1:20, 1:30, 1:40, 1:50, and 1:60 g/mL), the extraction power (311, 395, 479, 563, 647, and 731 W), pH (3, 4, 5, 6, and 7), the extraction temperature (30, 40, 50, 60, and 70 °C), and the extraction time (20, 40, 60, 80, 100, and 120 min) on the effect of the yield of the polyphenols. The product of the polyphenols under different single-factor conditions is shown in Figure 3. The results of the variance analysis show the ratio of the material to the liquid that had a significant effect on the yield of polyphenols (*p* < 0.01). The concentration of the ionic liquid and the extraction time had a substantial impact on the yield of polyphenols (*p* < 0.05), but other single factors had no significant effect.

(1) The effect of the mass fraction of ethanol. In Figure 3a, it can be seen that the 32 wt.% mass fraction of ethanol had the highest yield. Ethanol is an essential component of ethanol-potassium dihydrogen-phosphate-based aqueous two-phase systems. Ethanol and water can be mutually dissolved, and ethanol contains hydrophobic groups and hydrophilic groups. It can affect the aqueous two-phase system’s phase-separation ability and the target components’ yield [16]. As the mass fraction of ethanol increased, the ethanol-potassium phosphate system showed an increasing and then decreasing trend in the yield of polyphenols. The possible reason is that, when the mass fraction of potassium dihydrogen phosphate is 25 wt.% and the mass fraction of ethanol is 25 wt.%, the repulsive force generated by the hydrophobic group of ethanol and the phosphate group is such that the water molecules cannot balance the affinity of the hydrophilic group and the water molecules; as such, the phase cannot be separated [11]. When the mass fraction of ethanol is increased to 32 wt.%, the hydrophilic capacity increases, and the phase-separation capacity of the solvent system also increases. At the same time, the increase in polarity in the upper phase is beneficial to the dissolution of polyphenols and is enriched in the upper phase. When the mass fraction of ethanol is higher than 40 wt.%, the mass fraction of ethanol is too large, which increases the fat solubility of the upper phase, reduces the dissolution of polyphenols, and reduces the yield [17]. Therefore, the mass fraction of ethanol is best at 32 wt.%.
(2) The effect of the mass fraction of salts. Potassium dihydrogen phosphate is easily soluble in water. It exists in the system in an ionic form, which is a crucial component of the ethanol-potassium dihydrogen phosphate aqueous two-phase system [18]. In Figure 3b, with the increase in the mass fraction of the potassium dihydrogen phosphate, the yield of polyphenols in an ATPS first increases and then remains unchanged. The possible reason for this is that when the mass fraction of salt ions in the lower phase is low, the water molecules have a strong affinity for the hydrophilic groups of phosphate and ethanol; moreover, as the repulsive force between phosphate and ethanol is weak, it makes it impossible to separate the phases [18]. As the amount of salt gradually increases, the hydrophilicity of salt ions in the lower phase increases, thus promoting the system’s phase separation [19]. Therefore, changes in the amount of salt can affect the polarity of the upper phase, and ultimately, the distribution of the polyphenols therein. Thus, the mass fraction of salt was selected to be 25 wt.%.
(3) The effect of the IL-ATPS. The effects of the different imidazole IL-assisted ethanol/potassium dihydrogen phosphate systems on the yield of polyphenols are shown in Figure 3c. The yield of the polyphenols by the ATPS without adding IL, which was only 5.66 mg/g, is presented in Figure S1. The addition of IL improved the yield of polyphenols to varying degrees. This is because the upper phase was ethanol-enriched in the ethanol/KH_2_PO_4_ system, and the lower phase was salt-enriched. Polyphenols tend to be distributed in the more hydrophobic and low-charge ethanol-enriched phase. The effective extraction and enrichment of the target component by ATPS were mainly the result of the balance of various forces between the elements and the target components in the two phases. The driving forces include hydrophobic interaction, hydrogen bonding, electrostatic interaction, π–π bonding conjugation, and ionic liquid self-aggregation, which means that they can enhance this driving force, and they can promote the enrichment of the target component in the corresponding phase [20]. For example, the cation structure in IL can form hydrogen bonds with polyphenols, and the π electrons on the imidazole ring can be π–π-conjugated with the π electrons of the polyphenols, thus promoting the enrichment of polyphenols in the ethanol phase. In addition, when the carbon atoms of the alkyl chain of ionic liquid are more than 6, the ionic liquid will self-aggregate and increase the yield of the polyphenols. The anionic groups in IL can accept the protons in the polyphenols [21], which is conducive to the dissolution of polyphenols and to their enrichment by the ethanol phase—this also improves the yield of polyphenols [20]. The final choice of additional ionic liquid was 9 wt.%.
(4) The effect of the solid–liquid ratio. The data in Figure 3d, at the beginning, with the rise of the amount in the ATPS, show that the osmotic effect on *A. senticosus* powder was fully complete, and they also show that the increase in osmotic pressure will rupture the cell wall of *A. senticosus*, which will lead to the extraction of internal polyphenols. Finally, the water-phase solid–liquid ratio was 1:40 g/mL.
(5) The effect of ultrasonic power. The data in Figure 3e show that the product was at the highest when the ultrasonic energy was 396 W. This was due to the enhancement of ultrasonic power, such that the oscillation was strengthened, which is conducive to the extraction of polyphenols.
(6) The effect of ultrasonic temperature. The data in Figure 3f show that the polyphenol yield gradually increased when the ultrasonic temperature was increased from 30 °C to 50 °C. This was because with the rise in temperature, the average kinetic energy of the solvent molecules and the polyphenol molecules increased, the diffusion was enhanced, and the contact opportunities and times of the two molecules increased, thus increasing the number of polyphenols released. When the temperature exceeded 50 °C, the extraction rate of the polyphenols decreased. This was because the temperature was too high, and the solvent vaporization was serious, which hindered the continued dissolution of the polyphenols. Furthermore, it was found that a high temperature will accelerate the degradation of the phenolic substances. The optimum ultrasonic temperature was selected as 50 °C.
(7) The effect of the pH solution. The effect of the pH solution on the extraction of polyphenols is shown in Figure 3g. It can be concluded that under acidic conditions, the extraction rate of the polyphenols gradually increased with the increase of pH to 4. When the pH value grew, the extraction rate of the polyphenols evidently decreased. The pH value of the ATPS was adjusted by adjusting the amounts of hydrochloric acid and sodium hydroxide. The pH can influence the electrostatic interactions between solutes and solvents, thus significantly affecting the solute partitioning behavior in ATPSs [11]. When the solution is too acidic, it promotes the hydrolysis of polyphenols and reduces the yield. When the pH is >4, the alkalinity of the solution was enhanced, and the phenolic hydroxyl group of the polyphenols was readily dissociated into oxygen anions, thus making it undergo oxidative polymerization, which produces gelatinous substances and leads to a decrease in the polyphenol content in the extraction solution. This is due to the polyphenols’ structure, which is stable in a weakly acidic environment. Therefore, a pH of 4 was chosen.
(8) The effect of ultrasonic time. In Figure 3h, it can be seen that, when there was an increase in the ultrasonic time from 20 min to 120 min, the yield of the polyphenols increased. This occurred because the ultrasonic cavitation makes the polyphenols become more fully dissolved in the organic phase, thus increasing the yield of polyphenols within 20~40 min. On the other hand, the ultrasonic time was too short, and the efficiency of wall breaking was too low; as such, prolonging the ultrasonic time is beneficial in terms of increasing the yield of the polyphenols. When the ultrasonic time was over 40 min, the yield of polyphenols decreased. The possible reason for this is that when the ultrasonic time was 40 min, the polyphenols fully dissolved in the ethanol phase via the cavitation effect of the ultrasonic wave. When the ultrasonic time exceeded 40 min, the structure of the polyphenols was destroyed, and the hydroxyl group of polyphenols was oxidized; thus, the yield of polyphenols decreased. Therefore, an extraction time of 40 min was selected.

In conclusion, the optimal single-factor conditions for extracting *A. senticosus* polyphenols were as follows: the mass fraction of the ethanol at 32 wt.%, the mass fraction of the salt at 25 wt.%, the additional amount of ionic liquid at 9 wt.%, the solid–liquid ratio of *A. senticosus* and the double-water phase at 1:40 g/mL, and pH at 4.32. The extraction power was set at 395 W, the temperature was 50 °C, and the extraction time was 40 min.

### 2.4. Plackett–Burman Design

Based on the single-factor experiments, the Plackett–Burman design with *N* = 12 was used to investigate five factors: (A) extraction temperature, (B) extraction time, (C) extraction power, (D) the solid–liquid ratio, and (E) the amount of additional ionic liquid. High levels (1) and low levels (−1) were selected for each factor, and the yield of the polyphenols was taken as the response value. The design test and data processing were conducted with Design-Expert 12 software. The PB trial design factors and level codes are shown in Table 4, the trial results are represented in Table 5, and the analysis of variance results are provided in Table 6.

As shown by the data in Table 6, it can be concluded that the regression equation coefficient of the five factors with respect to the yield of the polyphenols delivered a significant test result. The effect of the solid–liquid ratio on the polyphenols’ extraction was highly significant (*p* < 0.01), and the impact of the additional ionic liquid and extraction time on the polyphenols’ extraction was also significant (*p* < 0.05). The order of significance was as follows: solid–liquid ratio > additional ionic liquid > extraction time. Therefore, the three factors with significant effects—extraction time (B), solid–liquid ratio (D), and additional ionic liquid (E)—were selected as the next step of the response surface model.

### 2.5. Box–Behnken Response Surface Methodology

Based on the results of the PB experiment, the ratio of the material-to-liquid, the amount of ionic liquid, and the extraction time were selected as the investigating factors. Moreover, the yield of the polyphenols was analyzed as the response value. Design-Expert 12 software was used for the response surface design. The design and results are shown in Table 7, and the analysis of variance results are shown in Table 8.

**Figure 4:**
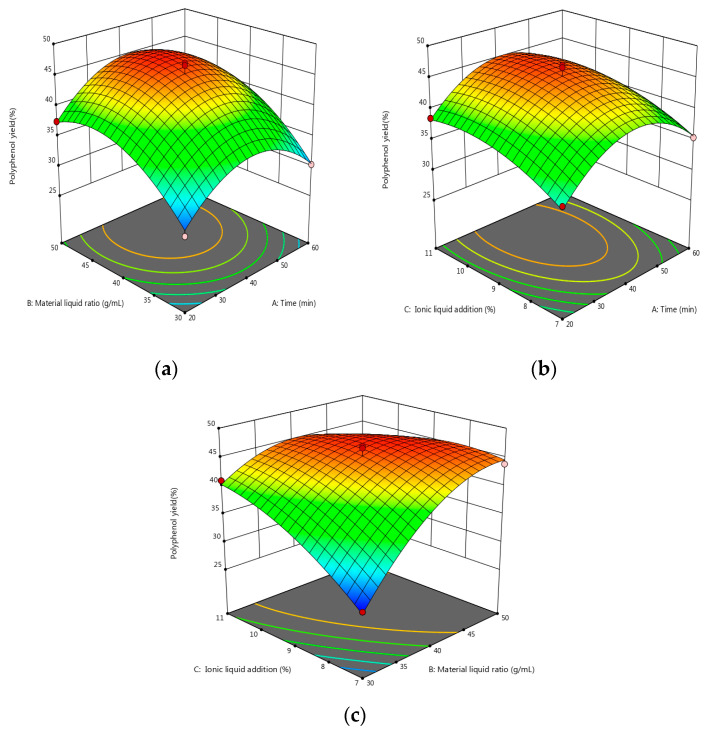
(**a**) Response surface map of the effects of extraction time and material–liquid ratio. (**b**) Response surface map of the effects of extraction time and ionic liquid addition. (**c**) Response surface map of the effect of material–liquid ratio and ionic liquid addition; the vertices indicate the maximum or minimum value of the response variable, while the points on the surface indicate the performance of this response variable at different levels of factors; the change from blue to red indicates a change from less to more the yield of polyphenols.

As shown by the data in Table 8, the model was statistically significant at *p* < 0.0001, and the coefficient of determination was R^2^ = 0.9784. This model can accurately reflect the influence of the various factors on the polyphenols’ extraction. When there was a correction factor of R^2^ = 95.06%, the simulation could account for 95.06% of the change in response. A lack of fit at *p* = 0.6731 was one of the factors not considered in the test as it had little influence on the test results. The CV was 3.46% < 10%, and the model fit well with the actual test. The signal-to-noise ratio was 17.2034. The model has good precision and a strong response signal. The above data show that the model can effectively predict the extraction of polyphenols. The visual graph was constructed to further explore the interaction between each factor and the relationship with the response value. Furthermore, the three-dimensional map of the model’s response surface was analyzed using Design-Expert 12 software (see Figure 4 for details).

As can be seen from Figure 4, the effects of the increase in the solid–liquid ratio and the amount of additional ionic liquid on the yield of polyphenols showed a trend of first increasing and then flattening. The extraction time tends to be gentle; as such, the polyphenol extraction rate can be improved by increasing the ratio of material-to-liquid, as well as the amount of ionic liquid.

According to the analysis of Design-Expert 12 software, the optimum extraction parameters were as follows: a solid–liquid ratio of 1:44.345 g/mL, and additional IL amount of 9.167 wt.%, and an extraction time of 41.744 min. Under these conditions, the extraction rate of the polyphenols was 44.142%. According to the actual situation and through considering the control range that each instrument can achieve in operation, the obtained theoretical factor parameters were modified as follows: the ratio of material-to-liquid was 1:45 g/mL, the amount of additional ionic liquid was 9 wt.%, and the extraction time was 42 min.

### 2.6. Validation Tests

The powder was weighed and treated with the optimized extraction parameters. The experiment was repeated three times. The yield of polyphenols was 43.246 mg/g, and the difference was 0.896% from the predicted value of the model. This model can be used to optimize the extraction process of polyphenols.

### 2.7. Optimization of the Macroporous Resin for the Purification of Polyphenols

#### 2.7.1. Selection of the Resins

**Figure 5:**
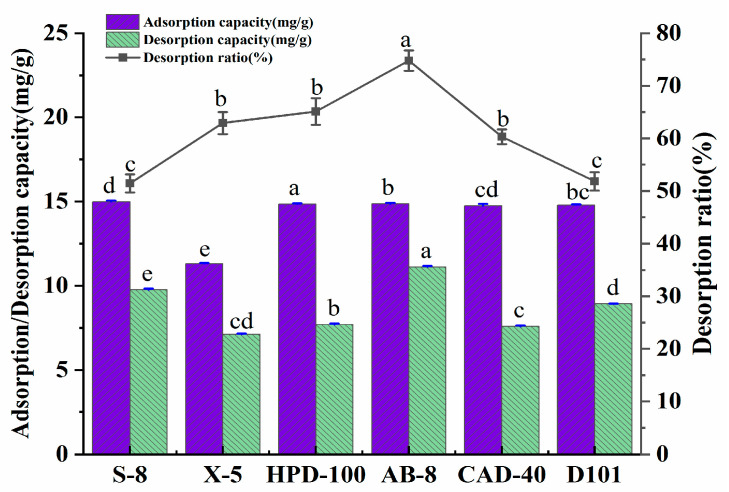
Adsorption/desorption amount (mg/g) and the desorption ratio (%) of *A. senticosus* polyphenols on the six different resins. Different letters represent significant differences (*p* < 0.05) between different vertical bars.

The appointable macroporous resin for the purification of the polyphenols was determined by exploring the adsorption amount, desorption amount, and the desorption rate of the six kinds of macroporous resins. The basic physical properties of the six macroporous resins are shown in Table 9. The results of the experiment are presented in Figure 5. Under the experimental conditions, the adsorption amounts of the macroporous resin on the polyphenols were in the order of: HPD-100 > AB-8 > D101 > CAD-40 > S-8 > X-5. The desorption amount of AB-8 resin was significantly higher than that of other resins.

The adsorption amount of the macroporous resin for the polyphenols was related to its polarity, specific surface area, and average pore size. Through the analysis that is shown in Figure 5, the polarity of the macroporous resin selected in this test had little effect on the adsorption amount of the polyphenols. In contrast, the specific surface area and average pore diameter significantly affected the adsorption amount of the polyphenols. The adsorption principle of the macroporous resin was that the molecular sieve formed by the porous network structure of the macroporous resin, as well as the high specific surface area, was used to adsorb the target substance. At the same time, the Van der Waals force and the hydrogen bonding force of the interaction between the resin and the target can also affect the adsorption amount of the resin. It is generally believed that the adsorption amount of a resin increases with the increase in the specific surface area. HPD-100 had the largest specific surface area among the six macroporous resins; thus, it has the largest adsorption capacity for the polyphenols. The specific surface area of S-8 was the smallest, but its adsorption amount for the polyphenols exceeded that of X-5, which indicates that adsorption is also related to the average pore diameter of the resin. Compared with the X-5 resin, the pore size of the S-8 resin was more favorable for the polyphenols to enter the resin. The desorption rate of AB-8 was the highest in terms of the desorption of the polyphenols. At the same time, that of HPD-100 was lower, which indicates that the pore size of HPD-100 was the lowest, suitable for the adsorption of polyphenols but not for their release. Therefore, the AB-8 resin was finally selected for the next step of the study when considering the best overall capacity for the adsorption amount, desorption amount, and desorption rate.

#### 2.7.2. Examination of the Concentration of the Adsorbent Solvent

**Figure 6:**
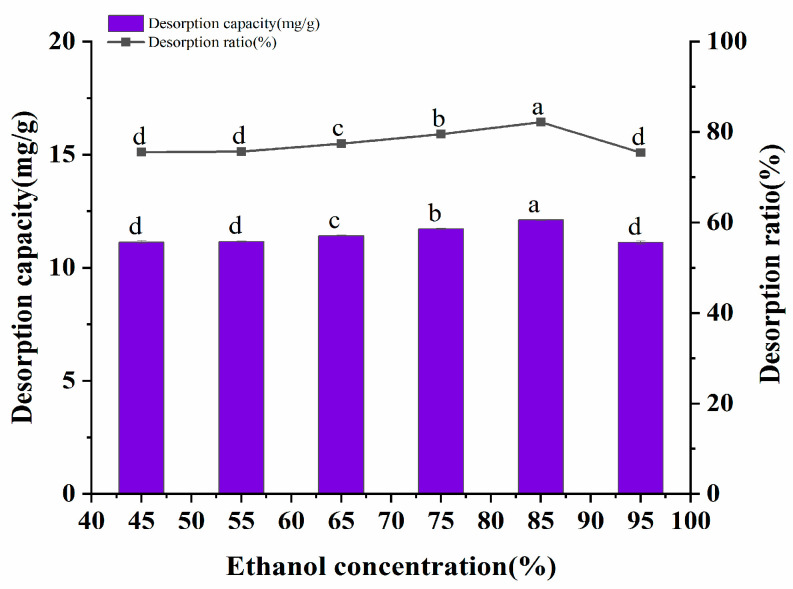
Effect of the solvent concentration on the static desorption rate of AB-8 resin. Different letters represent significant differences (*p* < 0.05) between different vertical bars.

It has been reported that polar solvents such as methanol, ethanol, and acetone have a higher desorption rate for polyphenols [22]. Ethanol was selected as the eluent due to consideration of the cost, among other reasons. The desorption amount and desorption rate of the polyphenols by different volume fractions of ethanol are shown in Figure 6. The desorption effect of 45~95% ethanol volume fractions on *A. senticosus* polyphenols was investigated in this experiment. The data represented in Figure 6 show that the desorption amount and desorption rate of the polyphenols increased with an increase in the ethanol volume fraction. When the volume fraction of ethanol exceeded 85%, the desorption rate of polyphenols decreased. This may be caused by the competitive dissolution of polyphenols with other alcohol-soluble substances, which are present due to the excessive volume fraction of ethanol. Therefore, 85% ethanol was selected as the eluent for the AB-8 resin.

#### 2.7.3. Dynamic Adsorption and Desorption Tests

**Figure 7:**
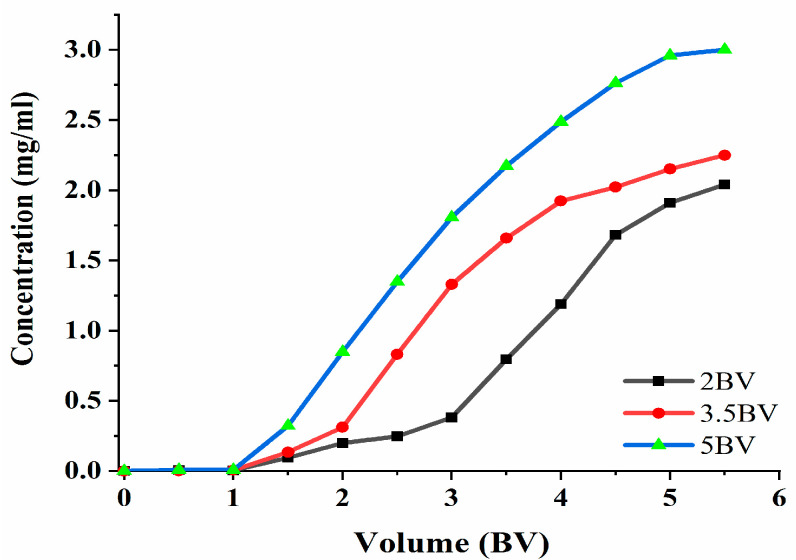
Leakage curve. Note: 1 BV = 20 mL, one tube per 10 mL.

The effluent concentration was 10% of the sample concentration. Furthermore, a leakage point was indicated [23], and adsorption can be terminated when the leakage point is present. The adsorption velocity has an influence on the adsorption capacity of the target; thus, the effect of the different adsorption velocities on the adsorption process was studied. The data in Figure 7 show that the polyphenol concentration in the effluent increased and then became slow, as can be seen under the three flow rates. The adsorption rate of the resin to the target decreased with the increase in the loading flow rate. The flow rate was 2 BV·h^−1^, 3.5 BV·h^−1^, and 5 BV·h^−1^, and the discharge point appeared when the effluent volume was 3 BV, 2 BV, and 1.5 BV. When the flow rate was 2 BV·h^−1^, the effluent concentration was the lowest, which indicated that the slower the flow rate, the more thoroughly the resin would adsorb the target; however, the slower the flow rate, the longer the amount of time taken. At 5 BV·h^−1^, the release point appeared the earliest, and the effluent concentration was the highest, thereby indicating that the polyphenol adsorption rate was the lowest. The reason for this may be that the contact time between the resin and the effluent was short, and that the resin could not reach saturation. The final choice was 3.5 BV·h^−1^, and the volume of the sample solution was set at 2 BV.

**Figure 8:**
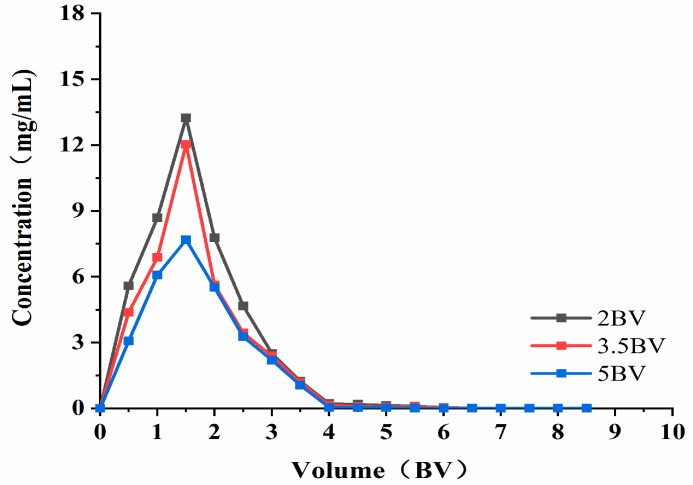
Elution curve. Note: 1 BV = 20 mL, one tube per 10 mL.

As can be seen from Figure 8, when the elution flow rate was 2 BV·h^−1^, the highest concentration of solution exited at 1.5 BV. When the elution flow rate was 3.5 BV·h^−1^, the attention of the effluent at 1.5 BV was the highest (which is similar to that found at 2 BV·h^−1^), and the elution peaks appeared more concentrated. This indicates that the target product can be eluted more thoroughly at lower flow rates. When the elution flow rate was 5 BV·h^−1^, the effluent concentration was lower than that of the low flow rate, and the peaks were not concentrated. The possible reason for this was that the elution speed was fast, and the eluent could not fully contact the resin to wash down the polyphenols adsorbed by the resin. As such, the final elution time and effect was set at 3.5 BV·h^−1^, and the volume used was 4 BV.

#### 2.7.4. Adsorption Isotherm

**Figure 9:**
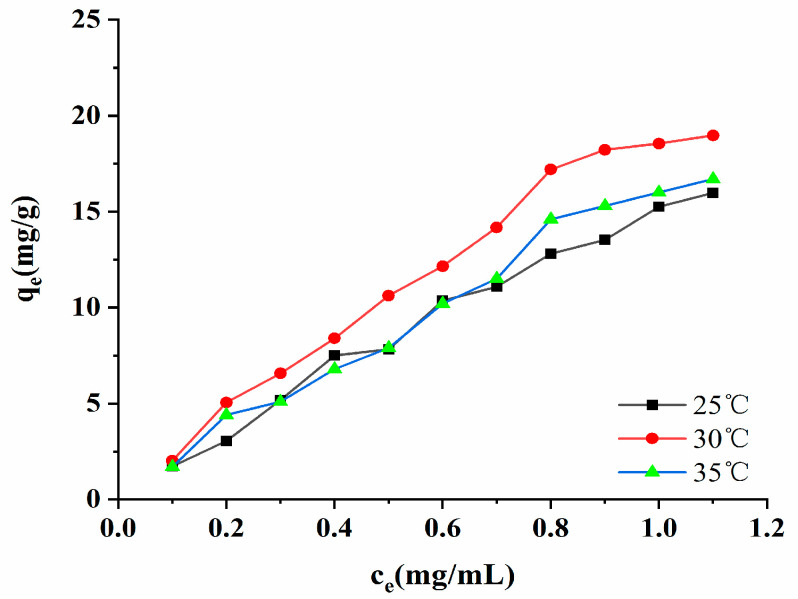
Adsorption isotherms.

When the macroporous resin adsorbed the target substance, the adsorption reached equilibrium. A fixed relationship existed between the target substance in the solution and the resin, and this is called an adsorption isotherm. The adsorption isotherm of the AB-8 macroporous resin is presented in Figure 9. As shown in Figure 9, when the temperature was the same, the concentration of the polyphenols was low, and the adsorption amount rapidly increased. When the concentration of the polyphenols reached 3.24 mg/mL, the adsorption tended to be gentle. When the adsorption amount does not change following an increase in the initial concentration of polyphenols, it indicates that the adsorption of the resin to the target substance has reached saturation [24]. The experimental data showed that the adsorption amount of the resin increased as there was an increase in temperature, thus indicating that the adsorption process is an endothermic reaction. The parameters of the Freundlich, Langmuir, and Temkin models are listed in Table S2. The test data indicated that the regression coefficient of the Freundlich model was the highest (R^2^ = 0.9295, 0.9817, 0.9853). It has been suggested that the Freundlich model is more suitable for evaluating the adsorption performance of AB-8 macroporous resin in absorbing the polyphenols. It was also able to show that the adsorption of the phenolic compounds by macroporous resin was due to multilayer adsorption and heterogeneous behavior [25]. The K_F_ of the model was related to the ability of the macroporous resin to adhere to the target, and the K_F_ value increased from 15.2549 (mg/g)(mL/mg)^1/n^ to 18.5506 (mg/g)(mL/mg)^1/n^ as the temperature rose from 25 °C to 30 °C. When the temperature increased from 30 °C to 37 °C, the K_F_ value decreased, indicating that the AB-8 macroporous resin had the best adhesion to polyphenols at 30 °C, and that it was the most favorable to resin adsorption. The exponential factor, n, in the model represents the non-homogeneous factor, and it is also an index through which to measure the degree of nonlinearity of the adsorption isotherm. The more significant the n, the better the adsorption effect of the resin. When 1 < *n* < 10, the adsorption was at its most favorable. When *n* < 1, the adsorption effect was deplorable, and this prevented the adsorption reaction from continuing [26].

The n parameters of the Freundlich model in this experiment were all greater than 1, indicating that the AB-8 macroporous resin easily adsorbed the polyphenols, as well as that the adsorption process involved a physical process [26].

### 2.8. Recycling of Ionic Liquid

#### 2.8.1. Ionic Liquid Decolorization and Ionic Liquid Retention

**Figure 10:**
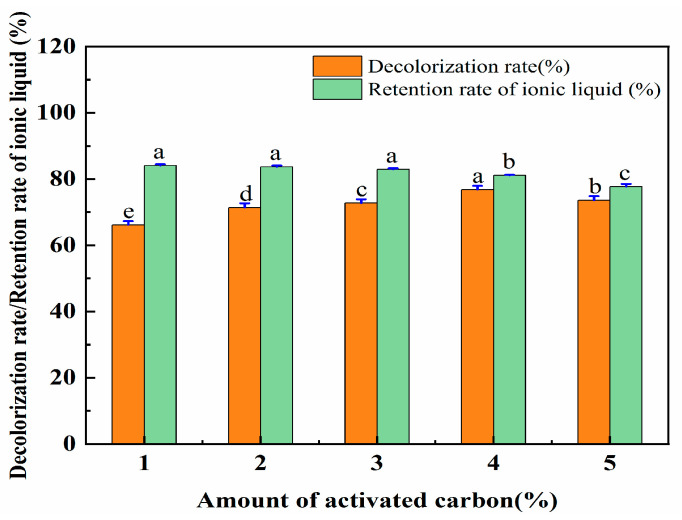
The effect of the amount of activated carbon on the decolorization rate and the retention rate of IL. Different letters represent significant differences (*p* < 0.05) between different vertical bars.

There are a large number of pores on the surface of activated carbon that can adsorb impurities. Therefore, the aqueous solution containing [OMIM]Br was treated with activated carbon. As the amount of activated carbon increased, the impurities were absorbed more thoroughly. However, when the amount of activated carbon exceeds a certain amount, it will be difficult to separate it from the water eluent contained in the solution. Increased amounts of activated carbon were used to adsorb and remove the contaminants. As such, a series of experiments were then conducted by using different amounts of activated carbon (1%, 2%, 3%, 4%, and 5%), which were utilized to ensure a high decolorization rate of the eluent and a high retention rate of the ionic liquid. The data for this are shown in Figure 10. The decolorization rate of the eluent increased with the increase in the amount of activated carbon. However, with the further increases in the activated carbon, the decolorization rate did not similarly significantly increase. For the retention rate of the IL, a slight reduction was followed when the amount of activated carbon was increased from 1% to 4%, and then a significant decrease when it was increased to 4–5%.

Regarding the decolorization rate of the eluent and the retention rate of the IL, an activated carbon amount of 4% performed better than the other amounts. Therefore, the activated carbon amount of 4% was selected for the treatment of the ionic liquid solution. The retention rate of the ionic liquid was set at 81.12%.

#### 2.8.2. Reuse of the [OMIM]Br Ionic Liquid

The recovered [OMIM]Br IL was reused to extract the polyphenols according to the conditions outlined in Section 2.2. Each experiment was carried out in triplicate. The recovery rate of the ionic liquid was 73.36%. The recovered ionic liquid was used to extract the polyphenols. The extraction efficiency of the polyphenols was 83.46%. The results shown indicate that the reuse rate of the ionic liquid was reasonable, and that it can be reused at least twice, which means that it can save the cost to a great extent. Moreover, the recovered [OMIM]Br ionic liquid also performed better for extracting polyphenols. This method was found to be effective for recovering and recycling [OMIM]Br.

## 3. Materials and Methods

### 3.1. Material

The *A. senticosus* powder (100 mesh) was obtained from the local pharmacy, and the macroporous resins of AB-8, S-8, D101, X-5, HPD-400, and CAD-40 (the six macroporous resins are all milky opaque spherical particles) were purchased from the Donghong Chemical Company, Qingdao, China.

The reagents used were as follows: gallic acid (AR ≥ 98.0%), 95% ethanol, sodium dihydrogen phosphate (NaH_2_PO_4_, purity ≥ 99%), sodium hydroxide (NaOH, purity ≥ 95%), hydrochloric acid (HCL, purity ≥ 99%), [C_4_mim]Cl, [C_4_mim]Br, [C_4_mim]BF_4_, [BMIM]OTF, [BMIM]SO_4_, [HMIM]Cl, [HMIM]BF_4_, [OMIM]BF_4_, and [OMIM]Br (purity ≥ 98%). The reagents were all purchased from Maclean’s (Shanghai, China), and the double-distilled water was prepared via a Milli-Q water purification system.

### 3.2. Instruments

A UV-5900 UV-Visible Spectrophotometer (190~1100 nm, glass cuvette, 10 mm) (Shanghai Yuanji Instrument Company, Shanghai, China), a BT100-2J Peristaltic Pump Driver (Baoding et al. Company, Baoding, China), a F-80 Portable Vacuum Pump (Tianjin Fufang Photoelectric Technology Development Company, Tianjin, China), a PE3000B Class Rotary Evaporator (Shanghai Yarong Biochemical Instrument Factory, Shanghai, China), a SB-600DTY ultrasonic multi-frequency cleaning machine (50~840 W, 25~59 Hz) (Ningbo et al. Company, Ningbo, China), and a HZQ-F160A thermostatic oscillator (Shanghai Yiheng Scientific Instrument Company, Shanghai, China) were all used in this study.

### 3.3. Optimization of the IL-ATPS’ Extraction of Polyphenols from A. senticosus

#### 3.3.1. Extraction Procedure of Polyphenols

In this paper, the feasibility of extracting and separating the polyphenols by adding the green solvent IL as an adjuvant into an ethanol-disodium hydrogen phosphate aqueous two-phase system, combined with the ATP extraction process, was investigated. Furthermore, 1 g of *A. senticosus* root powder and 2.5 g of sodium dihydrogen phosphate were accurately weighed and put into a 10 mL centrifuge tube. An amount of alcohol, water, and ionic liquid was added such that the mass fraction of the total system salt was 25 wt.%, the mass fraction of ethanol was 32 wt.%, the mass fraction of the ionic liquid was 5 wt.%, and the total volume was 10 mL. The mixture was stirred with a vortex mixer for a period of 10 min, thus forming two homogeneous phases. It was then placed in the ultrasonic cooker for a 40 min ultrasonic oscillation. After shaking to facilitate phase separation, the sample was centrifuged at 8000 rpm for 5 min. The polyphenols in the upper and lower phases of the double aqueous phase were determined by spectrophotometry.

#### 3.3.2. Determination of Polyphenols Content

A total of 0.5 mL of sample solution and 2.5 mL of Folin-Phenol reagent were added in a clean centrifugal tube. The tube was then placed in the dark for 8 min, and 2 mL of a 7.5% sodium carbonate solution was added, which was then kept in the dark for 1 h. The standard curve was performed by using gallic acid as a standard (the standard curve is shown in Figure S3), and the concentration of the polyphenols in the extract fluids was calculated according to the following standard curve:

y = 9.777x + 0.0398 R^2^ = 0.9953 (1)

#### 3.3.3. Screening of Aqueous Two-Phase System

The six different components of the ATPS were prepared and screened. The ingredients of the ATPS can be seen in Table 1. The yield of the polyphenols was used as the index to select the aqueous two-phase system. The calculation phase ratio (R), partition coefficient (K), and the yield of polyphenols (Y) were as follows:

(2) $$R=\frac{C_{t}V_{t}}{C_{t}V_{t}+C_{b}V_{b}}\;\times \;100$$

(3) $$K=\frac{C_{t}}{C_{b}}$$

(4) $$Y=\frac{C_{t}V_{t}}{M}$$

where R is the phase ratio (%), C_t_ is the concentrations of the polyphenols in the top phases (mg/mL), C_b_ is the concentrations of the polyphenols in the bottom phases (mg/mL), V_t_ is the separate volumes of the top phases (mL), V_b_ is the separate volumes of the bottom phases (mL), and M is the total mass of *A. senticosus* (g).

#### 3.3.4. Selection of the Ionic Liquids

Through the selection and combination of different anion and cation structures in the ILs, different hydrophilic and hydrophobic ILs can be obtained. These can control the phase secession behavior in the ATPS, as well as help in extracting and separating the active components, thus achieving the extraction or purification of various target components with differences in terms of physical and chemical properties. This paper investigated the effects of nine other ionic liquids (Table S1) in their capacity for extraction of the polyphenols. The polyphenols were enriched in the upper phase of the aqueous two-phase system.

#### 3.3.5. Ultrasonic-Assisted Extraction of the Polyphenols by IL-ATPS

The mass fraction of salt (21, 23, 25, 27, 29, and 31 wt.%), mass fraction of ethanol (30, 32, 34, 36, 38, and 40 wt.%), additional IL amount (3, 5, 7, 9, 11, and 13 wt.%), the solid–liquid ratio of IL-ATPS (1:10, 1:20, 1:30, 1:40, 1:50, and 1:60 g/mL), the extraction power (311, 395, 479, 563, 647, and 731 W), pH (3, 4, 5, 6, and 7), the extraction temperature (30, 40, 50, 60, and 70 °C), the extraction time (20, 40, 60, 80, 100, and 120 min), and the polyphenol yield were optimized as the evaluation indicators.

#### 3.3.6. Plackett–Burman Design

The Plackett–Burman design was used to investigate, according to the single-factor test, the significance of the influence factors on the extraction process of the polyphenols. In other words, five experimental factors were selected: extraction temperature (A), extraction time (B), extraction power (C), the solid–liquid ratio of IL-ATPS (D), and the additional IL amount (E). The factors were designed at high and low levels (1, −1), and the total score of the polyphenol yield was selected as the index. 

#### 3.3.7. Box–Behnken Response Surface Methodology

According to the PB test, the extraction time (B), the solid–liquid ratio of the IL-ATPS (D), and the additional IL amount (E) were selected as the factors to analyze. Moreover, the polyphenol yield was selected as the response value. The test results were designed according to Design-Expert 8.0.5.

### 3.4. Optimization of the Macroporous Resin for the Purification of Polyphenols from A. senticosus

#### 3.4.1. Pretreatment of the Macroporous Resin

The macroporous resin requires pre-processing before use. One should refer to the literature [27] for the specific method. Appropriate adjustments were made to the method according to the actual situation.

#### 3.4.2. Selection of the Resins

The optimal macroporous resin was screened with the adsorption amount, analytical amount, and desorption rate as indexes; in addition, 3 g of each of the six macroporous resins was prepared. A 20 mL, 3.24 mg/mL crude extract of the polyphenols was placed into a thermostatic oscillator and shaken for 24 h (at 30 °C and 150 rpm). The supernatant fluid was obtained by centrifugation at 4 °C and 1500 rpm. The resin was washed two times with distilled water and 20 mL of 90% ethanol was added, which was then shaken for 24 h (30 °C and 150 rpm). After 24 h, the supernatant fluids were centrifugated, and the absorbance was measured with a spectrophotometer. All experiments were performed in triplicate. The adsorption amount, desorption rate, and desorption amount were measured for the six resins, respectively, and the calculation formulas were as follows:

(5) $$q_{t}=\frac{(C_{0}-C_{1})\times V_{1}}{M}$$

(6) $$A=\frac{C_{2}\times V_{2}}{M}$$

(7) $$B=\frac{C_{2\times V_{2}}}{(C_{0}\times C_{1})\times V_{1}}\times 100\%$$

where q_t_ is the equilibrium adsorption amount per unit mass of the resin (mg/g), A is the desorption amount (%), B is the desorption rate (%), C_0_ is the mass concentration of the polyphenols before adsorption (mg/mL), C_1_ is the mass concentration of the polyphenols after adsorption (mg/mL), C_2_ is the mass concentration of the polyphenols after desorption (mg/mL), V_1_ is the sample liquid volume (mL), V_2_ is the desorption liquid volume (mL), and M is the dry mass of the macroporous resins (g).

#### 3.4.3. Examination of the Adsorbent Solvent Concentration

A total of 3 g of AB-8 resin was weighed, then the polyphenols were added. When the absorption reached adsorption saturation, it was put into a 50 mL conical flask. Then, 20 mL of an ethanol aqueous solution with volume fractions of 45, 55, 65, 75, 85, and 95% was added. Next, the flask was placed in a thermostatic water bath vibrator (at 30 °C and 150 rpm), then shaken for 24 h. The supernatant was then taken, and a measurement of the absorbance of the supernatant at a wavelength of 765 nm was conducted. Finally, the desorption rate of the AB-8 resin in an ethanol aqueous solution with different volume fractions was calculated.

#### 3.4.4. Dynamic Adsorption and Desorption Tests

We determined the volume of the loaded sample solution based on the following experimental conditions: The experiments were conducted on laboratory-scale glass columns (16 mm × 40 cm) that were wet-packed with 20 g of AB-8 resin. The total length of the resin bed was 20 cm. The IL-ATPS of the polyphenols flowed through the column at a rate of 3.5 BV/h. The effluent was collected in 1 BV (20 mL) glass sample tubes. The content of the polyphenols was determined in each line. The volume of effluent was as per the abscissa, and the concentration of the polyphenols in the effluent was as per the ordinate. A dynamic leakage curve was thus created.

A flow-rate resolution was obtained by loading the sample into the column first, followed by varying flow rates (1.5 BV/h, 3.5 BV/h, and 5 BV/h). Through using UV-vis absorption spectroscopy, the target compounds’ concentrations were measured.

According to the above conditions, dynamic adsorptions and desorptions were conducted. The 200 mL sample solutions flowed at 3.5 BV/h through the column. When the adsorption was organically saturated, the resin was washed using deionized water until the IL was no longer perceptible, via UV-vis absorption spectroscopy, in the eluant. The polyphenols were desorbed using a 95% ethanol volume fraction solution.

#### 3.4.5. Adsorption Isotherm

A total of 11 portions of 1 g of pretreated macroporous resin were accurately weighed into 100 mL conical flasks, which were then added to 25 mL of different concentrations of crude extract polyphenols (0.1, 0.2, 0.3, 0.4, 0.5, 0.6, 0.7, 0.8, 0.9, 1, and 1.1 mg/mL). These were then placed into a constant temperature shaker at 25 °C, 30 °C, and 35 °C (run at 100 rpm for 24 h). Thus, the polyphenol content of the supernatant after shaking at various concentrations was determined. The adsorption isotherms at different temperatures were obtained. To understand the adsorption mechanism of the AB-8 resin on the polyphenols, the adsorption isotherm was fitted linearly. The adsorption isotherm equation can quantitatively explain, at a constant temperature, the adsorption of the AB-8 resin on a crude extract of the polyphenols with different initial concentrations. In this paper, the most common Freundlich adsorption model (8), Langmuir adsorption model (9), and Temkin adsorption model (10) were used to fit the adsorption isotherms. To describe the adsorption mechanism, three adsorption isotherms were utilized as follows:

(8) $${\mathrm{lnq}}_{e}=\frac{1}{n}\;\cdot \;{\mathrm{lnC}}_{e}\;+\;{\mathrm{lnK}}_{F}$$

(9) $$\frac{C_{e}}{q_{e}}=\frac{1}{K_{L}q_{m}}+\frac{C_{e}}{q_{m}}$$

q_e_ = B_T_lnK_T_ + B_T_lnC_e_ (10)

where q_e_ is the equilibrium adsorption amount (mg/g), C_e_ is the equilibrium mass concentration of a solution (mg/mL), q_m_ is the saturated adsorption capacity (mg/g), K_F_ is the Freundlich equation constants ((mg/g)(mL/mg)1/n), n is the Freundlich equation constants (which can describe isotherm change trends), K_L_ is the Langmuir equation constants (which can be evaluated the amount of adsorption (mL/mg)), K_T_ is Temkin equation constants (mL/mg), and B_T_ is the Temkin equation constants (J/mol).

### 3.5. Recycling of the Ionic Liquid

Most of the IL could be washed with deionized water. In addition, a new ionic liquid solution with the same reference solution density was also used. By scanning with a spectrophotometer, the maximum absorption peak of the ionic liquid was determined, and this was located at 287 nm. Then, different bright activated carbon was added to the eluent, which was shaken for 1 h at 50 °C. As such, the absorbance of the eluent before and after decolorization was determined. The decolorization rate for the eluent was then calculated as follows:

(11) $$D_{c}=\frac{A_{0}-A_{1}}{A_{0}}\times 100\%$$

where D_c_ is the decolorization rate (%), A_0_ is the absorbance of the solution before treatment, and A_1_ is the absorbance of the solution after decolorization.

In addition, the retention rate of the IL in the resolution after decolorization was measured by UV-vis absorption spectroscopy. The discolored IL solution was evaporated at 95 °C for water recovery with a vacancy at −0.09 MPa, and was then dried for 2 h at 120 °C under a reduced pressure. The remaining liquid was the reclaimable IL, which was then reused for extraction.

### 3.6. Statistical Analysis

An analysis of variance was carried out using IBM SPSS 22.0 (SPSS Inc., Cary, NC, USA) software. The ANOVA was used for the statistical analysis of the data. Design-Expert version 7.0 was used to generate and analyze response surface and heat maps. Experiments were performed in triplicate, with the results expressed as the mean and standard deviation.

## 4. Conclusions

Ionic liquids are a new type of green solvent that was developed in recent years. It has a specific solubility to polar and non-polar solvents, and it has become a substitute for traditional solvents. ATP has an extraction function and a purification effect, which can improve the purity of the active components. An ionic liquid combined with two aqueous phases to produce a liquid with low volatility. With the advantages of high recovery, high balance, and easy amplification, it can effectively overcome the shortcomings of a single ionic liquid, and it can be used to extract multiple active ingredients [28]. The report of Gutowski et al. [29] also confirms this point; in their study, 1-butyl-3-methylimidazolium chloride and potassium phosphate (K_3_PO_4_) were first used. These have the advantages of a high extraction rate, low viscosity, and simple operation. Almeida et al. [9] added an ionic liquid to a polyvinyl alcohol/sodium sulfate aqueous two-phase system, which resulted in a significant improvement in the extraction ability of gallic acid.

In this paper, the enrichment and extraction of the polyphenols from *A. senticosus* were accomplished by using an ionic liquid as an adjuvant in an ethanol/NaH_2_PO_4_ hydrogen phosphate aqueous two-phase system. The adsorption and desorption properties of AB-8 were better, and an 85% volume fraction of ethanol was used. The Freundlich model was more suitable for the equilibrium adsorption experiment. Moreover, it was found that resin adsorption is the preferential adsorption. In a study by Wang et al. [13,19], the purity of epigallocatechin gallate crystallized by LX-20B macroporous resin resulted in a 95.87 ± 0.89% and 95.55 ± 1.30% extraction rate. Moreover, the resin was reused six times, thus indicating that the macroporous resin used had a good reuse capability.

An activated carbon amount of 4%, a decolorization rate of 76.81%, and an ionic liquid retention rate of 81.12% performed best. The recovery rate of the ionic liquid was 73.36%. The extraction rate was higher, and the reuse rate of the ionic liquid was also higher under these conditions.

## Figures and Tables

**Table 1:** Screening of aqueous two-phase systems: composition and phase-forming capability of the systems.

ATPS	Concentration of Bottom Phase (wt.%)	Concentration of Water (wt.%)	Phase Demixing and Formation Feature
ATPS1	NaH_2_PO_4_ 25	47	Fast, Easy, Stable, Transparent
ATPS2	(NH_4_)_2_SO_4_ 25	47	Fast, Easy, Stable, Transparent
ATPS3	NaH_2_PO_4_ 25	47	Fast, Easy, Stable, Transparent
ATPS4	(NH4)_2_SO_4_ 25	47	Fast, Easy, Stable, Transparent
ATPS5	NaH_2_PO_4_ 25	47	Fast, Easy, Stable, Transparent
ATPS6	(NH4)_2_SO_4_ 25	47	Fast, Easy, Stable, Transparent

**Table 2:** Screening of aqueous two-phase system: phase ratio (R), partition coefficient (K), extraction rate (Y, %), and polyphenol yield (%).

ATPS	Phase Ratio (R)	Partition Coefficient (K)	Extraction Rate (Y, %)	Polyphenol Yield (mg/g)
Ethanol/NaH_2_PO_4_	3.44	7.04	96.03	5.66
Ethanol/(NH4)_2_SO_4_	0.73	8.02	85.42	4.29
PEG400/NaH_2_PO_4_	3.82	5.12	95.14	5.32
PEG400/(NH4)_2_SO_4_	0.77	16.5	92.7	4.84
Acetone/NaH_2_PO_4_	0.86	10.23	89.8	4.53
Acetone/(NH4)_2_SO_4_	0.41	20.66	89.51	3.39

**Table 3:** Ionic liquids: phase ratio (R), partition coefficient (K), extraction rate (Y, %), and polyphenol yield (%).

ILs	Phase Ratio (R)	Partition Coefficient (K)	Extraction Rate (Y, %)	Polyphenol Yield (mg/g)
[C_4_mim]Cl	4.111	16.865	98.58	6.29
[C_4_mim]Br	3.747	11.969	97.82	6.50
[C_4_mim]BF_4_	3.330	12.166	97.59	5.94
[BMIM]OTF	3.002	8.474	96.22	6.14
[BMIM]SO_4_	4.105	9.851	97.59	6.02
[HMIM]Cl	3.680	5.568	95.35	2.40
[HMIM]BF_4_	3.258	7.732	96.18	3.62
[OMIM]BF_4_	2.780	26.719	98.67	13.01
[OMIM]Br	2.923	25.337	98.67	15.90

**Table 4:** Variables and their coded levels used in the Plackett–Burman design.

Level	Factors				
	Temperature (A)/°C	Time (B)/min	Power (C)/W	Solid–Liquid Ratio (D)/g/mL	IL Addition Amount (E)/(g, *w*/*w*)
−1	40	20	311	30	7
1	60	60	479	50	11

**Table 5:** Design of experiment for screening (Plackett–Burman design).

Test Group	Temperature (A)/°C	Time (B)/min	Power (C)/W	Material–Liquid Ratio (D)/g/mL	IL Addition Amount (E)/ (wt.%)	Polyphenol Yield (%)
1	1 (60)	−1 (20)	−1 (311)	−1 (30)	1 (11)	37.231
2	1 (60)	1 (60)	−1 (311)	1 (50)	1 (11)	22.712
3	1 (60)	−1 (20)	1 (479)	1 (50)	1 (11)	14.533
4	−1 (40)	1 (60)	1 (479)	−1 (30)	1 (11)	41.047
5	−1 (40)	1 (60)	−1 (311)	1 (50)	1 (11)	16.091
6	1 (60)	1 (60)	−1 (311)	−1 (30)	−1 (7)	30.688
7	1 (60)	−1 (20)	1 (479)	1 (50)	−1 (7)	10.511
8	−1 (40)	−1 (20)	−1 (311)	−1 (30)	−1 (7)	28.141
9	−1 (40)	−1 (20)	−1 (311)	1 (50)	−1 (7)	10.316
10	−1 (40)	1 (60)	1 (479)	1 (50)	−1 (7)	10.706
11	1 (60)	1 (60)	1 (479)	−1 (30)	−1 (7)	36.61
12	−1 (40)	−1 (20)	1 (479)	−1 (30)	1 (11)	32.078

**Table 6:** Analysis of variance of the Plackett–Burman model.

Source	Sum of Squares	df	Mean Square	F-Value	*p*-Value
Model	1399.34	5	279.87	32.52	0.0003 **
A	16.11	1	16.11	1.87	0.2202
B	52.27	1	52.27	6.07	0.0488 *
C	0.0078	1	0.0078	0.0009	0.9770
D	1218.59	1	1218.59	141.59	<0.0001 **
E	112.36	1	112.36	13.06	0.0112 *
Residual	51.64	6	8.61		
Cor. Total	1450.98	11			

* Significant at *p* < 0.05. ** Significant at *p* < 0.01.

**Table 7:** Box–Behnken test design and results.

Coded Values	Test Group	Time (B)/min	Material–Liquid Ratio (D)/g/mL	IL Addition Amount (E)/(%, *w*/*w*)	Polyphenol Yield (%)
9	1	40	30	7	27.8226
12	2	40	50	11	40.3325
13	3	40	40	9	45.0575
4	4	60	50	9	41.0274
11	5	40	30	11	40.9907
15	6	40	40	9	46.9714
2	7	60	30	9	30.3256
14	8	40	40	9	44.0575
1	9	20	30	9	28.5844
10	10	40	50	7	43.8745
17	11	40	40	9	46.5976
6	12	60	40	7	35.4115
3	13	20	50	9	37.4957
16	14	40	40	9	43.5878
8	15	60	40	11	39.0067
5	16	20	40	7	33.8205
7	17	20	40	11	38.537

**Table 8:** Analysis of variance of the Box–Behnken model.

Source	Sum of Squares	df	Mean Square	F-Value	*p*-Value
Model	576.29	9	64.03	35.20	<0.0001 ***
A: Time	6.72	1	6.72	3.70	0.0960
B: Material–liquid ratio	153.18	1	153.18	84.22	<0.0001 ***
C: IL addition amount	40.22	1	40.22	22.11	0.0022 **
AB	0.8015	1	0.8015	0.4406	0.5281
AC	0.3143	1	0.3143	0.1728	0.6901
BC	69.81	1	69.81	38.38	0.0004 **
A^2^	163.35	1	163.35	89.81	<0.0001 ***
B^2^	91.73	1	91.73	50.43	0.0002 **
C^2^	22.89	1	22.89	12.59	0.0094 **
Residual	12.73	7	1.82		
Lack of Fit	3.73	3	1.24	0.5527	0.6731
Pure Error	9.00	4	2.25		
Cor. Total	589.03	16			

** Significant at *p* < 0.01. *** Significant at *p* < 0.0001.

**Table 9:** Physical properties of the employed macroporous resins.

Resin	Surface Area (m^2^/g)	Average Pore Diameter (Å)	Particle Diameter (mm)	Polarity	Moisture (%)
AB-8	450~530	13~14	0.3~1.25	Middle-polar	62~72
S-8	100~120	280~300	0.3~1.25	Polar	66~72
X-5	500~600	290~300	0.3~1.25	Non-polar	55
D101	500~550	9~10	0.3~1.25	Non-polar	65~75
HPD-100	650~700	85~90	0.3~1.20	Non-polar	65~75
CAD-40	450~500	50~60	0.25~0.84	Middle-polar	55~65

## Data Availability

Not applicable.

## Supplementary Materials

The following supplementary materials can be downloaded at: https://www.mdpi.com/article/10.3390/molecules28176383/s1, Table S1: Ionic liquids and solubility. Table S2: Adsorption isotherm models fitting regression equations and adsorption parameters at different temperatures. Figure S1: Different structural formulas of ionic liquids. Figure S2: Decolorization effect of activated carbon. Figure S3: Standard curve for polyphenol content.

## Sample Availability

Samples of the compounds are available from the authors.
