# Supplementary material for: Study on Extraction and Purification of Acanthopanax senticosus Polyphenols by an Ionic Liquid-Assisted Aqueous Two-Phase System
Source: Molecules. 2023 Aug 31;28(17):6383. doi: 10.3390/molecules28176383 (PMC10490139; doi:10.3390/molecules28176383)
Supplement: Supplementary file 1 [file molecules-28-06383-s001.zip › molecules-2580255-supplementary.pdf]

Study on Extraction and Purification of *Acanthopanax senticosus* Polyphenols by an Ionic Liquid-assisted Aqueous Two-phase System

Ying Li [1], Xiaoli Li [2], Xueyan Wang [3], Jiaojiao Xue [4], Rui Zhang [5], Yi Ding [6], Xiuling Chu [2,*] and Jianqing Su [2,*]

[1]Shandong Provincial Institute of Chinese Veterinary Medicine, Liaocheng University ,Liaocheng 252000,China

*Correspondence: chuxiul@163.com (X.C.); sujianqing@lcu.edu.cn (J.S.)

Supporting Information

Table S1. Ionic liquids and solubility.

ILs number	Abbreviation	Full name	Solubility in water
IL1	[C4mim]Cl	1-Butyl-3-methylimidazole chloride	Totally miscible
IL2	[C4mim]Br	1-Buthyl-3-methylidazolium bromide	Totally miscible
IL3	[C4mim]BF4	1-Butyl-3-methylimidazole tetrafluoroborate	Totally miscible
IL4	[BMIM]OTF	1-Butyl-3-methylimidazolium trifluoromethanesulfonate	Totally miscible
IL5	[BMIM]SO4	1-Butyl-3-methylimidazolium hydrogen sulfate	Totally miscible
IL6	[HMIM]Cl	1-Hexyl-3-methylimidazolium chloride	Totally miscible
IL7	[HMIM]BF4	1-Hexyl-3-methylimidazolium tetrafluoroborate	Totally miscible
IL8	[OMIM]BF4	1-Octyl-3-methylimidazolium tetrafluoroborate	Totally miscible
IL9	[OMIM]Br	1-Octyl-3-methylimidazolium Bromide	Totally miscible

we have conducted experiments on the solubility of ionic liquids: an amount of alcohol, water, ionic liquid was added that the mass fraction of ethanol was 32 wt. %, the mass fraction of the ionic liquid was 3, 5, 7, 9, 11, and 13 wt.%, and the total volume is 10 mL, stir at room temperature to form a homogeneous and transparent solution, which will not crystallize when stored at room temperature for 24h.

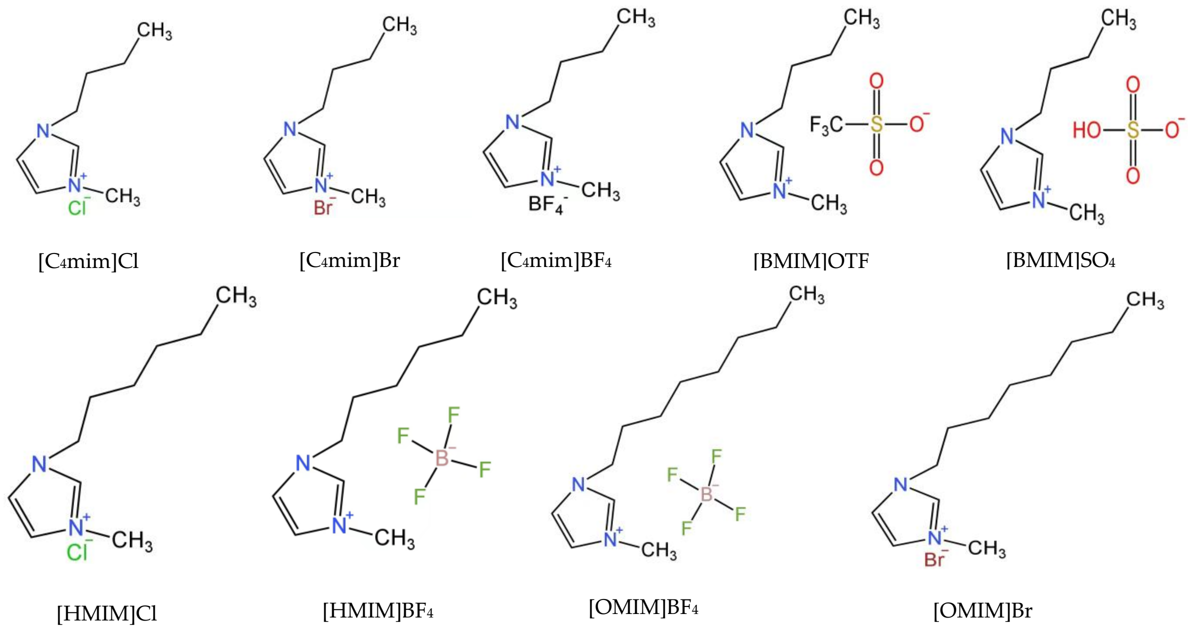

Figure S1. Different structural formulas of ionic liquids.

Table S2. Adsorption isotherm models fitting regression equations and adsorption parameters at different temperatures.

Model	T/(° C)	Equation	Parameter		
			K_F[(mg/g)(mL/mg)$^{1/n}$]	n	R^2
Freundlich	25	$\ln C_e$=0. 9465Ce+2. 7249	15. 2549	1. 0565	0. 9295
	30	$\ln C_e$=0. 9619Ce+2. 9205	18. 5506	1. 0396	0. 9817
	37	$\ln C_e$=0. 9854Ce+2. 7726	16. 0000	1. 0148	0. 9853
			k_L(mL/mg)	Q_m(mg/g)	R^2
Langmuri	25	C_e/q_e=0. 084Ce+0. 0575	0. 1469	118. 4210	0. 3913
	30	C_e/q_e=0. 0050Ce+0. 0489	0. 1022	200. 0000	0. 5241
	37	C_e/q_e=0. 0041Ce+0. 0549	0. 0749	243. 2432	0. 2781
			K_T(L/mg)	B_T(J/mol)	R^2
Temkin	25	q_e=5. 9448lnCe+15. 4143	13. 3631	5. 9448	0. 935
	30	q_e=7. 0687lnCe+18. 3016	13. 3178	7. 0687	0. 9358
	35	q_e=6. 2555lnCe+16. 1042	13. 1234	6. 2555	0. 9073

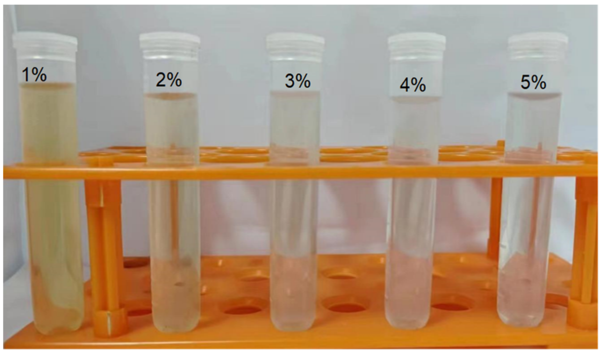

Figure S2. Decolorization effect of activated carbon.

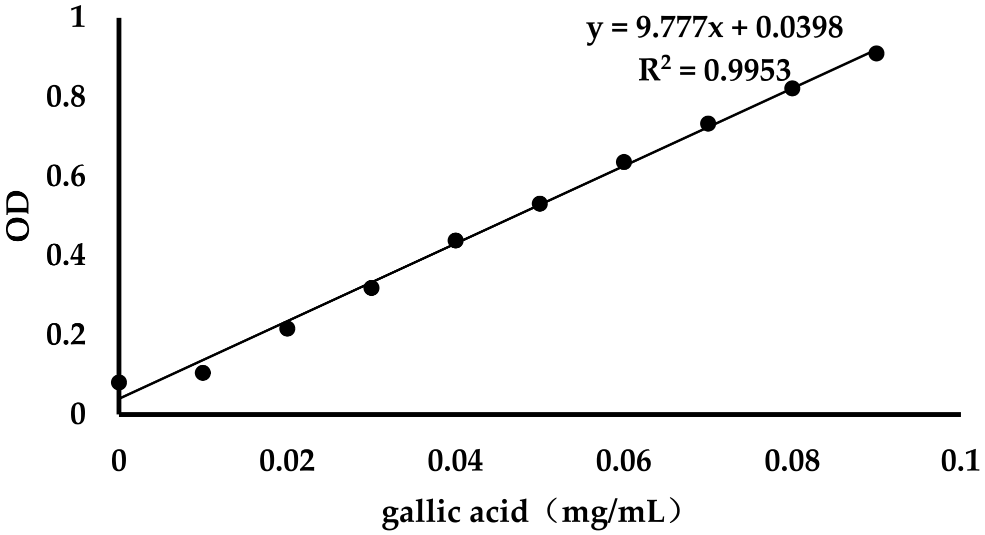

Figure S3. Standard curve for polyphenol content.
